# Pathogenicity of *Mycolicibacterium phlei, a* non-pathogenic nontuberculous mycobacterium in an immunocompetent host carrying anti-interferon gamma autoantibodies: a case report

**DOI:** 10.1186/s12879-019-4050-z

**Published:** 2019-05-22

**Authors:** Satomi Tanaka, Yoshihiko Hoshino, Takuro Sakagami, Hanako Fukano, Yohei Matsui, Osamu Hiranuma

**Affiliations:** 1 Otsu City Hospital, 2-9-9, Motomiya, Otsu-city, Shiga Prefecture Japan; 2 Leprosy Research Centre, National Institute of Infectious Diseases, 4 Chome-2-1 Aobacho, Higashimurayama, Tokyo, Japan; 3 Department of Respiratory Medicine, Faculty of Life Sciences, Kumamoto University, 1-1-1 Honjo, Chuo-ku, Kumamoto, Japan

**Keywords:** Disseminated infection, *M. phlei*, Autoantibody, Interferon gamma

## Abstract

**Background:**

*Mycolicibacterium phlei* (*M. phlei*) is known to be a non-pathogenic nontuberculous mycobacterium (NTM) which rarely causes diseases in humans. A disseminated NTM infection is mostly caused by the *Mycobacterium avium* complex (MAC) and is known to develop in immunocompromised hosts, like those with acquired immune deficiency syndrome (AIDS). Here, we report a case of disseminated *M. phlei* infection in an immunocompetent host carrying anti-interferon gamma (IFN-γ) autoantibodies.

**Case presentation:**

We detected *M. phlei* in multiple organs of an elderly woman with no significant medical history except positivity for anti-IFN-γ autoantibodies. She tested negative for human immunodeficiency virus (HIV)-1, 2/ Human T-cell leukemia virus type 1 (HTLV-1) antibody. High-resolution computed tomography (HRCT) of the chest demonstrated a nodule in the left S1 + 2 segment, interlobular septal thickening, multi lymphadenopathy, and osteolysis. A maximum intensity projection image following fluorodeoxyglucose-positron emission tomography (FDG-PET) revealed multifocal hypermetabolic lesions in the nodule and all the swollen lymph nodes seen in HRCT. FDG also accumulated in multiple bones. Advanced primary lung cancer was suspected, and biopsies of each lesion were performed. The pathology revealed caseating granuloma, positive for acid-fast bacteria, and DNA sequencing of the acid-fast bacteria confirmed the organism to be *M. phlei*. The patient also tested positive for anti-IFN-γ autoantibodies. Based on these findings, she was diagnosed with disseminated *M. phlei* infection, with anti-IFN-γ autoantibodies.

**Conclusions:**

Though known to be non-pathogenic, we show that *M. phlei* can be pathogenic like the MAC in immunocompetent individuals carrying anti-IFN-γ autoantibodies.

## Case report

### Background

Disseminated nontuberculous mycobacterium (NTM) infection is a disease that primarily occurs in immunocompromised hosts, such as those with acquired immune deficiency syndrome (AIDS). However recently some reports on disseminated NTM infection in immunocompetent hosts have been published, and most of them carry anti-interferon gamma (IFN-γ) autoantibodies [1]. Almost all of these cases of NTM involve *Mycobacterium avium* complex (MAC) or other pathogenic NTM.

*Mycolicibacterium Phlei* (*M. phlei*) is classified as a non-pathogenic NTM [1]. While there are very few reports of *M. phlei* causing infections in humans [2–8], there are none of it causing disseminated infections. Based on its non-pathogenicity, *M. phlei* has been used for the treatment of conditions like asthma [9].

Here, we report a case of disseminated *M. phlei* infection in an immunocompetent host carrying anti-IFN-γ autoantibodies. It is possible that the pathogenicity of *M. phlei* in such hosts is different from that in hosts with a normal immune system.

## Case presentation

**Fig. 1:**
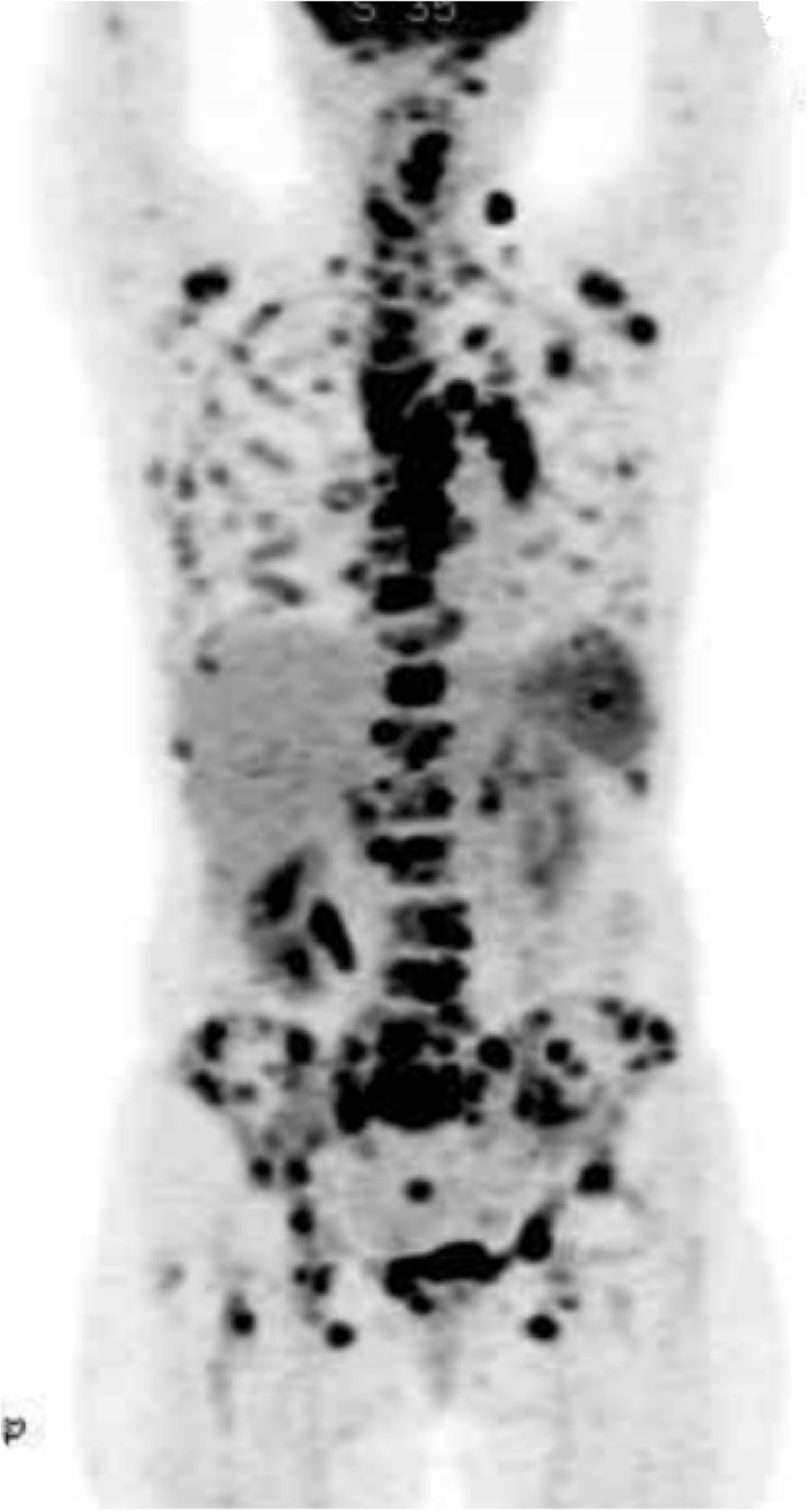
A maximum intensity projection image following fluorodeoxyglucose-positron emission tomography (FDG-PET) revealed multifocal hypermetabolic lesions in the nodule in the left S1 + 2 segment, interlobular septal thickening in the left lower lobe, lymphadenopathy of the left hilar, mediastinal, supraclavicular, and posterior cervical lymph nodes. FDG also accumulated in the anterior spinal cord, sacrum, iliac bone, pubic bone, ischium, sternum, scapula, ribs, clavicle, and thigh bone

A 79-year-old woman with no significant medical history visited the Otsu City Hospital (Shiga prefecture, Japan) complaining of chest pain that had persisted for the last one month. Her vital signs were normal, and except for diffuse erythema with lichenification of the trunk and limbs, the physical examination was unremarkable. The initial laboratory data indicated a high level of inflammation with a white blood cell count (WBC) of 18,100 /μL (normal range: 3800–9400 /μL), c-reactive protein (CRP) levels of 7.56 mg/dL (0.00–0.03 mg/dL), and an erythrocyte sedimentation rate (ESR) of 136 mm/h (1.0–15.0 mm/h). She tested negative for human immunodeficiency virus (HIV)-1, 2/ Human T-cell leukemia virus type 1 (HTLV-1) antibody. QuantiFERON-Gold in tube ^®^ (QFT) yielded indeterminate results. She tested negative for MAC infection, based on the serum levels of anti-glycopeptidolipid-core immunoglobulin (Capilia MAC Antibody ELISA)^®^ [10]. The levels of immunoglobulin G (IgG), IgA, IgM were normal (Table 1). High-resolution computed tomography (HRCT) of the chest demonstrated a nodule in the left S1 + 2 segment, interlobular septal thickening in the left lower lobe, lymphadenopathy of the left hilar, mediastinal, supraclavicular, and posterior cervical lymph nodes, and osteolysis of the sternum and the left second rib, which might have caused the chest pain. She also had hepatosplenomegaly. A maximum intensity projection image following fluorodeoxyglucose-positron emission tomography (FDG-PET) revealed multifocal hypermetabolic lesions in the nodule and all the swollen lymph nodes seen in HRCT. FDG also accumulated in the anterior spinal cord, sacrum, iliac bone, pubic bone, ischium, sternum, scapula, ribs, clavicle, and thigh bone (Fig. 1).

**Table 1:** The laboratory data, initial and 2 years after

Hematology (initial)			Biochemistry (initial)					
WBC	18,100	/μL	TP	6.9	g/dL	CEA	2.6	ng/mL
Neut	69.5	%	Alb	2.8	g/dL	SCC	0.9	ng/mL
Eos	16	%	AST	9	U/L	NSE	10.3	ng/mL
Lym	11	%	ALT	16	U/L	ProGRP	33.9	pg/mL
Hb	8.9	g/dL	LDH	192	U/L	CYFRA	1.6	ng/mL
Ht	27.2	%	ALP	522	U/L	CA19–9	5	U/mL
MCV	86.3	μm3	γ-GTP	79	U/L	sIL-2R	10,252	U/mL
MCHC	32.7	%	CK	16	U/L	HTLV-1 antibody	< 16	times
Plt	375,000	/μL	Na	138	mEq/L	T-SPOT^®^	(−)	
ESR	136	mm/h	K	4.5	mEq/L	QuantiFERON^®^	< 0.05	
			Cl	102	mEq/L			undeterminate
(2 years after)			BUN	23	mg/dL	Capillia MAC IgA^®^	(−)	
			CRE	0.93	mg/dL	IgG	1072	mg/dL
WBC	7400	/μL	Blood Sugar	106	mg/dL	IgA	144	mg/dL
Lym	20.4	%	CRP	7.56	mg/dL	IgM	61	mg/dL
CD4	30.7	%	PCT	0.43	ng/mL	HIV-1/2 antibodies	(−/−)	
CD8	39.7	%	β-D glucan	< 2.4	pg/mL			
CD4/CD8	0.8					PHA SI	528.4	

*PCT* procalcitonin, *CEA* carcinoembryonic antigen, *SCC* Squamous Cell Carcinoma, *CYFRA* cytokeratin 19 fragment, *NSE* nerve specific enolase, *Pro GRP* pro-gastrin releasing peptide, *CA19–9* carbohydrate antigen 19–9, *sIL-2R* soluble interleukin-2 receptor, *PHA SI* the lymphocyte phytohemagglutinin (PHA) stimulation index

Magnetic resonance imaging (MRI) of the lumbar to thoracic vertebrae was performed. Based on a T2-weighted image showing a mottled abnormal signal, bone marrow infiltration or necrosis was suspected.

Advanced primary lung cancer was suspected, and a cervical lymph node biopsy was performed. The pathology revealed a caseating granuloma, positive for acid-fast bacteria. We performed the left iliac bone marrow and transbronchial lung biopsies (TBLB). While the bone marrow biopsy revealed tiny granulomas, no special findings were seen in TBLB. Since there was no evidence of malignancy, video-assisted thoracic surgery was performed for confirmation. The nodule in the left S1 + 2 segment on HRCT could not be identified directly, and therefore, biopsies of the para-aortic, swollen lymph node and pleura were performed instead. The biopsies revealed epithelioid granuloma, which stained positive for acid-fast bacteria. The real time -polymerase chain reaction was negative for tuberculosis or MAC. The number of acid-fast bacteria on the biopsy specimens was so low that no pathogen was detected after their culture. From these results, it was concluded that she had disseminated NTM infection, even though she had no history of being immunocompromised except for the indeterminate QFT^®^ results.

**Fig. 2:**
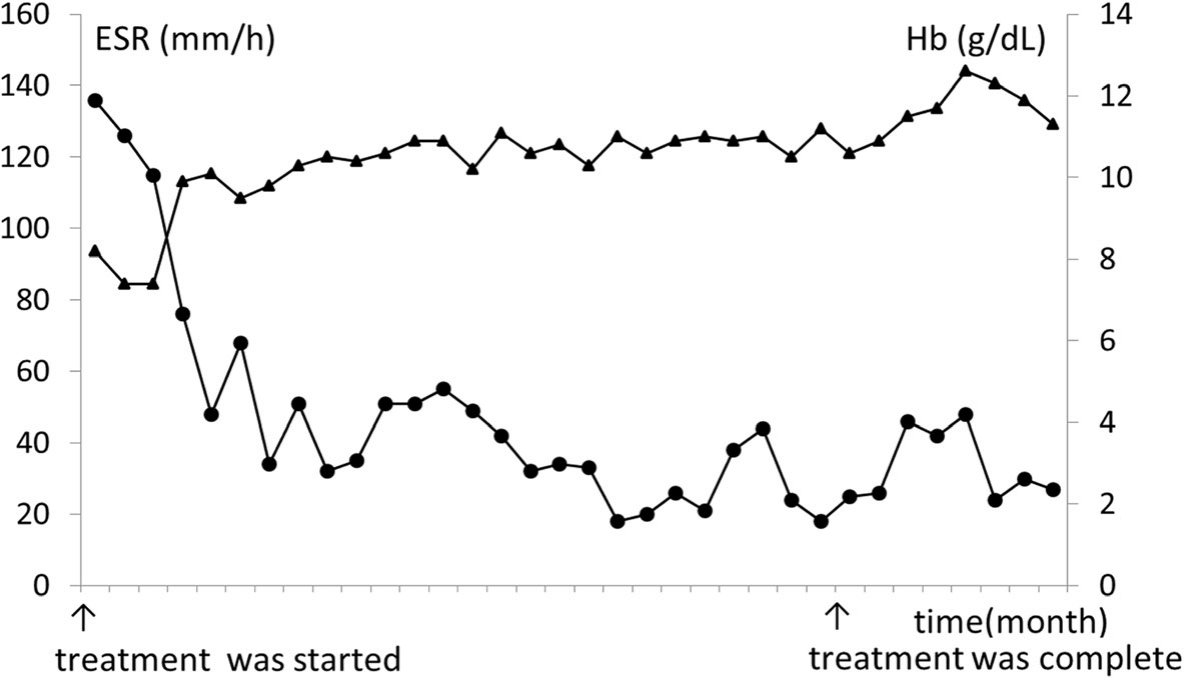
Following the completion of treatment in 26 months, her ESR levels normalized

She was started on a multi-drug combination therapy which included rifampicin 300 mg/day, ethambutol 500 mg/day, clarithromycin 600 mg/day, and levofloxacin 300 mg/day. Because the sputum test was negative for mycobacterium based on the Ziehl–Neelsen staining, the follow up was based on her ESR and CRP levels, both of which improved gradually. Since we did not culture blood for acid-fast bacteria and no pathogens were detected following the culture of the biopsy specimens, we recovered the genome of the bacteria from the paraffin blocks. The partial nucleotide sequences of the *hsp65* and *rpoB* genes were amplified and sequenced. DNA sequencing confirmed the organism to be *M. phlei*. We concluded she had IFN-γ autoantibody, because her serum retained high concentration of neutralizing capacity to IFN-γ, measured by flow-cytometry based method and the antigen capture assay formerly described [11]. Based on these results, we evaluated the levels of CD4 and CD8, as well as the lymphocyte phytohemagglutinin (PHA) stimulation index to assess her cell-mediated immunity. She had 30.7% (463/μL) CD4 (normal range: 25.0–54.0%) and 39.7% CD 8 (23.0–56.0%). The CD4:CD8 ratio was 0.8 (0.40–2.30), and the lymphocyte PHA stimulation index was 528.4 (101.6–2643.8). Her cell-mediated immunity was normal. Following the completion of treatment in 26 months, her ESR levels normalized. The swelling in the lymph nodes and the abnormal vertebral lesions showed improvement. Following-ups are ongoing (Fig. 2).

## Discussion and conclusions

Anti IFN-γ autoantibodies are associated with adult-onset of immunodeficiency akin to that seen in patients with acquired immunodeficiency syndrome (AIDS). These antibodies can cause disseminated NTM infections in an immunocompetent host, which was previously known to occur only AIDS patients [1]. The presence of these antibodies has been associated with disseminated NTM mostly caused by pathogenic NTM, except in the case of *M. mantenii* infections, the virulence of which has never been clearly demonstrated [12].

*M. phlei* belongs to the Runyon’s classification group IV of rapidly growing potential pathogens [13], but the American Thoracic Society does not list it as a pathogenic mycobacterium [14]. The molecular basis for the virulence of the mycobacterium species is still not well understood. Studies on *M. phlei* by Beltan et al. [15] have suggested that important differences exist between pathogenic and non-pathogenic mycobacterium species, particularly with respect to TNF-α and GM-CSF signalling. Raynaud et al. [16] have reported the role of extracellular enzymes in the pathogenicity of mycobacterium species. Twenty-two enzyme activities were detected from the culture fluids and/or cell surfaces of mycobacterium species, of which eight were absent from the culture fluids of non-pathogens. They have also suggested that the molecular architecture of the mycobacterial envelopes may play an important role in the pathogenicity of these organisms. These studies support the non-pathogenicity of *M. phlei*. Based on its non-pathogenicity in humans, *M. phlei* has been used for the treatment of conditions like asthma. The inhalation of it can reduce airway inflammation by regulating IL-4, IL-10, and IFN-γ secretion [9]. Reports on *M. phlei* infections are extremely rare, with eight cases reported as per our search (Table 2) [2–8]. The case reported here is the ninth case, but the first one to describe a disseminated form of the infection. Almost all of the reported infections were in immunocompetent hosts, except for the eighth one wherein the patient had AIDS. However, in this case, the patient had a normal X-ray, and the sputum test was positive for *M. phlei* only once, suggesting mere colonization rather than a pathogenic role.

**Table 2:** Reports on *M. phlei* infections

No. [Reference]	Age /Gender	Infected Organs	Background	Immuno- suppressive drug	Medication	Duration of therapy/ clinical outcome
1. [2]	7/M	synovial fluid and tissue	healthy	none	INH, RFP, and SM	9 month/cured
2. [3]	49/M	flexor digitorum longus and posterior tibialis tendon of his right foot.	healthy	none	CAM and CPFX	2 month/cured
3. [4]	17/M	peritonitis	CAPD due to FSGS	none	AMK, CFX and DOXY	9 month/cured
4. [5]	73/F	pacemaker infection	ICM(NYHAIII), DM, HT	none	DOXY	12 month/cured
5. [6]	N/A	lung	healthy	none	N/A	N/A
6. [6]	N/A	lung	healthy	none	N/A	N/A
7. [7]	N/A	lung	N/A	N/A	N/A	N/A
8. [8]	35/M	None (colonization)	AIDS CD4 455/μL	N/A	N/A	N/A
This case	79/F	Lung, lymph nodes, multiple bones	anti-IFN-γ autoantibodies	none	RFP, EB, CAM, and LVFX	26 month/cured

*N/A* not available, *CAPD* Continuous ambulatory peritoneal dialysis, *FSGS* focal and segmental glomerulosclerosis, *ICM* Ischemic cardiomyopathy, *NYHA* New York Heart Association, *DM* diabetes mellitus, *HT* hypertension, *AIDS* Acquired immune deficiency syndrome, *INH* Isoniazid, *RFP* Rifampin, *SM* Streptomycin, *CAM* Clarithromycin, *CPFX* Ciprofloxin, *AMK* Amikacin, *CFX* Cefoxitin, *DOXY* Doxycycline, *EB* Ethambutol, *LVFX* Levofloxacin

The patient in our study also had no significant medical or drug history, except positivity for anti-IFN-γ autoantibodies. To understand why this non-pathogenic mycobacterium could cause disseminated infection in immunocompetent individuals, we focused on IFN-γ dependent immunity, which is essential for the control of mycobacterial infections. For example, Mendelian susceptibility to mycobacterial disease (MSMD), that leads to paediatric chronic diseases, is a rare condition characterized by susceptibility to weakly virulent mycobacteria, such as Bacille de Calmette et Guérin (BCG) vaccines and environmental mycobacteria [17]. Though MSMD was ruled out in our patient based on her age and the absence of any significant medical history, it is possible that her IFN-γ dependent immunity might have been impaired as in an MSMD patient. More detailed studies using the patient’s blood cells are needed to confirm this possibility.

Therefore, to the best of our knowledge, our case is the first report suggesting the pathogenicity of *M. phlei* and its ability to cause a disseminated “non-pathogenic” NTM infection in hosts carrying anti-IFN-γ autoantibodies. However, what causes an individual to have anti-IFN-γ autoantibodies is not clear. If it is related to environmental mycobacterial infections, its clinical significance could be much higher because *M. phlei* is also used for the treatment of conditions such as asthma.

## Abbreviations

QFT: QuantiFERON-Gold in tube ^®^
AIDS: Acquired immune deficiency syndrome
AMK: Amikacin
BCG: Bacille de Calmette et Guérin
CA19–9: Carbohydrate antigen 19–9
CAM: Clarithromycin
CAPD: Continuous ambulatory peritoneal dialysis
CEA: Carcinoembryonic antigen
CFX: Cefoxitin
CPFX: Ciprofloxin
CRP: C-reactive protein
CYFRA: Cytokeratin 19 fragment
DM: Diabetes mellitus
DOXY: Doxycycline
EB: Ethambutol
ESR: Erythrocyte sedimentation rate
FDG-PET: Fluorodeoxyglucose-positron emission tomography
FSGS: Focal and segmental glomerulosclerosis
HIV: Human immunodeficiency virus
HRCT: High-resolution computed tomography
HT: Hypertension
HTLV-1: Human T-cell leukemia virus type 1
ICM: Ischemic cardiomyopathy
IFN-γ: Interferon gamma
IgG: Immunoglobulin G
INH: Isoniazid
LVFX: Levofloxacin
*M. phlei*: *Mycolicibacterium phlei*
MAC: *Mycobacterium avium* complex
MRI: Magnetic Resonance Imaging
MSMD: Mendelian susceptibility to mycobacterial disease
N/A: Not available
NSE: Nerve specific enolase
NTM: Non-pathogenic nontuberculous mycobacterium
NYHA: New York Heart Association
PCT: Procalcitonin
PHA SI: The lymphocyte phytohemagglutinin (PHA) stimulation index
PHA: Phytohemagglutinin
Pro GRP: Pro-gastrin releasing peptide
RFP: Rifampin SM Streptomycin
SCC: Squamous Cell Carcinoma
sIL-2R: Soluble interleukin-2 receptor
TBLB1: Transbronchial lung biopsies
WBC: White blood cell count

## Authors’ information

Nothing.
